# Prenatal Environment That Affects Neuronal Migration

**DOI:** 10.3389/fcell.2019.00138

**Published:** 2019-07-17

**Authors:** Hye M. Hwang, Ray Y. Ku, Kazue Hashimoto-Torii

**Affiliations:** ^1^Center for Neuroscience Research, Children’s National Medical Center, The Children’s Research Institute, Washington, DC, United States; ^2^The Institute for Biomedical Sciences, School of Medicine and Health Sciences, The George Washington University, Washington, DC, United States; ^3^Departments of Pediatrics, and Pharmacology & Physiology, School of Medicine and Health Sciences, The George Washington University, Washington, DC, United States

**Keywords:** prenatal environmental stress, neuronal migration, neuronal migration disorders, fetal brain development, Cxcl12/Cxcr4 signaling, heat shock signaling

## Abstract

Migration of neurons starts in the prenatal period and continues into infancy. This developmental process is crucial for forming a proper neuronal network, and the disturbance of this process results in dysfunction of the brain such as epilepsy. Prenatal exposure to environmental stress, including alcohol, drugs, and inflammation, disrupts neuronal migration and causes neuronal migration disorders (NMDs). In this review, we summarize recent findings on this topic and specifically focusing on two different modes of migration, radial, and tangential migration during cortical development. The shared mechanisms underlying the NMDs are discussed by comparing the molecular changes in impaired neuronal migration under exposure to different types of prenatal environmental stress.

## Introduction

*In utero* environment critically affects the brain development, thereby modifying neurobehavior of the offspring after birth (Rees and Harding, 2004). Substances that are ingested by a pregnant woman can cross the placenta and adversely affect fetal development. Some are unavoidable medications due to the mother’s medical condition, such as epilepsy or depression, but others are consumed by an expectant mother unbeknown to the risks and effects. In some cases, an excessive amount may be taken by a pregnant woman due to the addiction.

**FIGURE 1:**
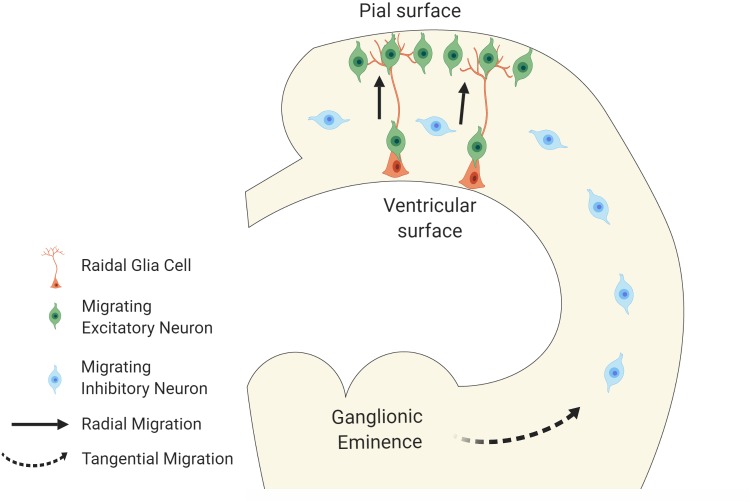
Modes of neuronal migration in cortical development. The schematic diagram illustrates two major modes of migration. Excitatory neurons migrate radially from ventricular surface toward the pial surface of the neocortex using radial glial cells as their scaffolds whereas the inhibitory neurons migrate tangentially from the ganglionic eminences to the neocortex.

During brain development, newly generated neurons undergo morphological changes followed by migrating from the germinal layer through the intricate network of extracellular matrix to establish connections with other cells in a highly ordered fashion (Rakic, 1972, 1995; Metin et al., 2008). Two major modes of migration in the cerebral cortex are known (Copp and Harding, 1999; Guerrini and Parrini, 2010). The first is radial migration. Excitatory neurons generated in the proliferative zones of the dorsal telencephalic primordium radially migrate toward the pial surface of the cerebral cortex along the radial axis. These neurons use the cellular processes that are elongated from the radial glial cells, which are ascending neural progenitor cells (NPCs), as the scaffold of their migration (Rakic, 1972; Ayala et al., 2007; Figure 1). Once arriving at the superficial layer, the neurons detach from the radial glial processes to settle down in the cortical plate. Another mode of neuronal migration, known as tangential migration, is employed by inhibitory interneurons. Those neurons migrate tangentially from the ganglionic eminences (GE) to the cerebral cortex (Marín and Rubenstein, 2001; Ayala et al., 2007; Figure 1).

Any disturbances of these two modes of migration cause neuronal migration disorders (NMDs), and the consequent malformations are detectable by brain imaging in NMD patients (Roberts, 2018). Profound cases of NMDs include lissencephaly, heterotopia, and focal dysplasia (Guerrini and Parrini, 2010; Roberts, 2018). Precise control of the migration and positioning of both excitatory and inhibitory neurons are particularly important for the formation of synaptic excitation and inhibition (E/I) balanced circuit in the brain. Therefore the E/I imbalance is the main cause of epilepsy in NMD patients (Copp and Harding, 1999). Other neurodevelopmental and psychiatric disorders, including autism spectrum disorders (ASD), and schizophrenia are also associated with NMDs (Canitano and Pallagrosi, 2017).

In addition to the genetic causes, harmful prenatal environment also leads to neuronal migration defects (Metin et al., 2008). When developmental neurotoxicology first emerged, various paradigms of exposure were deployed in many species to recapitulate human pathophysiology, including defects of neuronal migration. Decades later, many recent studies have utilized genetic tools and cutting edge molecular techniques in those preclinical animal models to decipher the underlying mechanisms of neuronal migration defects under the stress exposure. In this review, we summarize such recent findings in animal research and discuss the mechanisms shared by various types of environmental stress as the targets of interventions.

## Alcohol

Prenatal alcohol exposure (PAE) is the cause of fetal alcohol spectrum disorders (FASD) (Jones and Smith, 1973; Burd et al., 2007; Riley et al., 2011; Wilhoit et al., 2017). Alcohol consumed by a pregnant woman can easily cross the placenta, and increases fetal blood alcohol concentrations to the levels equivalent to those in maternal side within 2 h of ingestion (Burd et al., 2012). Affected individuals of FASD display a spectrum of defects, including facial malformations and neurobehavior impairments (Subramoney et al., 2018).

Depending on the timing of exposure to alcohol and the mother’s drinking pattern, alcohol may have different effects on a child (Foltran et al., 2011). A comprehensive transcriptome study of mice that were exposed to two acute doses (5 g/kg in total) of alcohol at various periods of brain development demonstrated that ethanol exposure at different developmental periods impacts differential biological processes (Kleiber et al., 2013). Notably, the genes that are associated with cell migration and differentiation were altered only in the brains exposed to alcohol on E14 and E16 which is equivalent to second trimester in humans (Kleiber et al., 2013).

Neuronal migration defects have been reported by many groups in animal models of PAE. In a binge-drinking model in which the mice were exposed to 5% w/w alcohol at E13.5–E16.5, defects of radial migration particularly affecting the populations of excitatory neurons were found in the somatosensory cortex (Delatour et al., 2018; Table 1). These animals also showed a decrease of dendritic complexity in excitatory neurons and reduced tactile sensitivity (Delatour et al., 2018). In another study using the same binge drinking paradigm (5% w/w E13.5–E16.5 exposure), the increase of interneurons derived from medial ganglionic eminence (MGE) in the medial prefrontal cortex (mPFC) was observed by using Nkx2.1Cre-Ai14 transgenic mice. This increase of interneurons in the mPFC is due to the increase of proliferation of the progenitor cells in MGE as well as earlier entrance of those neurons into the mPFC (Skorput et al., 2015). Associated E/I imbalance that favors synaptic inhibition was also reported (Skorput et al., 2015; Table 1). Chronic liquid administration of ethanol at lower dosage (1 or 2% w/v) from early to mid-gestational stages (E0.5–E14.5) increased the number of inhibitory interneurons in the neocortex(Cuzon et al., 2008; Table 1).

**Table 1:** Summary of different environmental exposure paradigm studies described in this review.

Type of defect	Exposure paradigm				Model			
	Type	Dose and route	Exposure time in animal	Equivalent human trimester	Species	Age of assessment	References
Radial migration defect	Ethanol	5% w/w, self -administered Liber-DeCarli liquid diet regimen	E13.5–E16.5	Second trimester	Thy1-YFP Tg mice	E16.5	Delatour et al., 2018
	Hypoxia	7% oxygen for 8 min/h for 10 h per day	E2–E20	First and second trimester	Sprague-Dawley rats	P6	Zechel et al., 2005
		7% oxygen for 3 h per day	E14 or E18	Second trimester	Wistar rats	P5	Vasilev et al., 2016
		9% oxygen for 2 h per day	E13.5		C57BL/6J or Balb/cBYJ mice	E14	Herr et al., 2011
	Methylmercury	0.01,0.1 or 1 mg/kg/day, IP injection	E11–E21	First and second trimester	Sprague-Dawley rats	P0, P3 and P7	Guo et al., 2013
	Glucocorticoids	200 μg/kg of DEX, daily SC injection	E14.5–E20.5	Second trimester	Wistar rats	E16.5–E21.5	Fukumoto et al., 2009
Tangential migration defect	Ethanol	1 or 2 % w/v, self-administered liquid diet regimen (Dam BAL 28.94 ± 1.97 mg/dL at E13.5)	E0.5–E14.5	First and second trimester	GAD67-GFP knock-in mice	E14.5	Cuzon et al., 2008
		5% w/w, self-administered liquid diet regimen (Dam BAL 80 ± 21 mg/dL at E15.5)	E13.5–E16.5	Second trimester	Nkx2.1-Ai14 (tdTomato) Tg mice	P70	Skorput et al., 2015
	Glucocorticoids	Stress induced GC, by restraint stress three times a day	E12–P0		GAD67-GFP knock-in mice	E13, E14, E15 and P0	Stevens et al., 2013
Radial and tangential migration defect	Cocaine	20 mg/kg, SC injection twice a day	E8–E12, E13 or E15	First and second trimester	Swiss Webster GAD67-GFP knock-in mice	E12, E13, and E15	McCarthy et al., 2011
Migration speed defect	Selective serotonin reuptake inhibitor (SSRI)	400 μM, 90 min exposure, cortical slices	E17.5 and P0.5	Second trimester	C57BL/6 mice	E17.5 and P0.5	Riccio et al., 2011
	Ethanol	10 and 50 mM, acute exposure on cerebellar slices	P7 and P13	Third trimester	CD1 mice	P7 and P13	Kumada et al., 2006
	Methylmercury	Various of dose between 0.01 and 5.0 μg/g body weight	P6–P9		CD1 mice	P10	Fahrion et al., 2012
Ectopic localization	Maternal Immune Activation (MIA)	6000 plague-forming units (PFU) of human influenza virus one time IP injection or 20 mg/kg of poly(I:C) one time IP injection	E9.5 or E12.5	First trimester	Balb/c mice	P11	Shi et al., 2009
	Valproic acid (VPA)	300 mg/kg, oral administration once a day	E12–E14	Second trimester	C57BL/6 mice	P84	Sakai et al., 2018
	Cocaine	20 mg/kg, IP injection twice a day	E13–E14, E15–E16		Sprague-Dawley rats	E15 and E17	Lee et al., 2011


*BAL, blood alcohol level; IP, intraperitoneal; SC, subcutaneous.*

Intracellular Ca^2+^ serves as a second messenger of various signaling, and dynamic oscillation of the intracellular Ca^2+^ is associated with neuronal migration (Komuro and Rakic, 1996). Interestingly, Kumada et al. reported the migration defect observed in cerebellar granule cells following ethanol exposure *ex vivo* were rescued by modifying the Ca^2+^ signaling (Table 1). The authors demonstrated that exposure to ethanol reduces the cGMP level, but increases the cAMP level in the tissue, resulting in changes of Ca^2+^ transients in the migrating neuron. Importantly, the neuronal migration defects were reversed by stimulating the brain with Ca^2+^ and cGMP or inhibiting cAMP signaling both *in vitro* and *in vivo* (Kumada et al., 2006). Rescuing neuronal migration through changing Ca^2+^ and cyclic nucleotide signaling is mediated by activating the downstream targets that are essential for neuronal migration, such as protein kinase C (PKC), Ca^2+^/calmodulin-dependent protein kinase II (CaMKII), protein phosphatase 1 (PP1), Rho GTPase, mitogen-activated protein kinase (MAPK) and phosphoinositide 3-kinase (PI_3_K) (Huang et al., 2004; Shmueli et al., 2006; Zhao et al., 2009; Khodosevich and Monyer, 2011).

As mentioned earlier, alcohol exposure changes the expressions of many genes that are associated with neuronal migration. Transcriptome analyses of human and mouse fetal cerebral cortices that were acutely exposed to alcohol demonstrated altered expressions of genes that are related to neuronal migration (Hashimoto-Torii et al., 2011; Kleiber et al., 2013; Kawasawa et al., 2017). Of note, a study on the expressions of splicing isoforms in the human brain revealed global exon skipping following alcohol exposure (Kawasawa et al., 2017). Consequently, the functions of the encoded proteins may be different before and after alcohol exposure. However, how the changes in expressing protein isoforms lead to migration deficiency, and the mechanism of alcohol leading to global changes in RNA splicing remains to be investigated.

## Cocaine

In human, prenatal cocaine exposure (PCE) is associated with changes in cortical structure, physical and neurocognitive development in the offspring (Bellini et al., 2000; Richardson et al., 2015). The association between cocaine exposure in second trimester and poor motor development in the infants based on psychomotor developmental index (PDI) was reported in a cohort study (Richardson et al., 2008). However, given that cocaine users frequently abuse other drugs and substances, the consequences of PCE at different stages of the trimester is not clearly defined (Martin et al., 2016).

Although animal studies clearly show that PCE in early developmental stages induces a more severe effects in neuronal migration than that in late developmental stages (Lee et al., 2011; McCarthy et al., 2011). The animals subjected to cocaine at 20 mg/kg twice a day during early/mid gestation periods (E13–E14 or E15–E16) showed similar levels of accumulation of postmitotic excitatory neurons in the ventricular zone of the cerebral cortex (Lee et al., 2011; Table 1). This indicates that the migration deficits can be explained by indirect effects due to changes in the proliferation and differentiation of NPCs in the germinal zone.

Disruption of C-X-C motif chemokine ligand 12 (CXCL12)/C-X-C chemokine receptor type 4 (CXCR4) pathway is another potential mechanism of the neuronal migration defects that are elicited by cocaine exposure. Reduction of CXCR4 protein expression following exposure to cocaine was reported by Hu et al. (2006). They exposed human fetal brain-derived NPCs to cocaine *in vitro* and observed inhibition of cellular proliferation and directional migration toward CXCL12 as well as reduction of CXCR4 expression (Hu et al., 2006). Similarly, the positioning of the interneurons that express CXCR4 in upper cortical layers was disturbed in *Cxcr4* knockout mice, providing additional evidence that CXCR4 plays a role in neuronal migration (Stumm et al., 2003). However, neither study replenishes CXCR4 to ameliorate the neuronal migration defects. This piece of data will strengthen the crucial role of CXCR4/CXCL12 signaling in neuronal migration, and indicate the potential of the signaling as a target of therapeutic intervention for NMDs.

McCarthy et al. (2011) proposed that the reduction of brain-derived neurotropic factor (BDNF) post exposure to cocaine may also explain impaired migration of the interneurons (Table 1). The animals that were injected with 20 mg/kg of cocaine twice a day show defects not only in radial migration of excitatory neurons but also in tangential migration of inhibitory neurons (McCarthy et al., 2011). These defects were associated with a transient decrease in BDNF protein expression (McCarthy et al., 2011). BDNF plays a pivotal role in neurogenesis in the developing cerebral cortex, and its interaction with CXCR4 has been reported (Ohmiya et al., 2002; Bachis et al., 2003). In fact, Xu and Heilshorn (2013) demonstrated that BDNF significantly enhances the directional migration of human NPCs toward CXCL12. Furthermore, the presence of BAMD3100, a blocker of CXCR4, completely abolishes the migration, suggesting that BDNF-mediated chemotaxis toward CXCL12 is through CXCR4 activation (Xu and Heilshorn, 2013). Although how BDNF and CXCR4 interact during neuronal migration is still unclear, BDNF-mediated CXCL12/CXCR4 signaling may critically contribute to the impairment of neuronal migration under cocaine exposure.

## Hypoxia

Intrauterine hypoxia occurs when the fetus is deprived of an adequate supply of oxygen. The inadequate oxygen level may be caused by high altitude and pre-existing maternal cardiovascular diseases, including heart failure and pulmonary hypertension. Maternal anemia, infections, chronic inflammation, and smoking also limit the amount of oxygen delivered to the fetus (Hutter et al., 2010). Chronic hypoxia during brain development affects radial and tangential migrations of excitatory and inhibitory neurons, respectively, thereby leading to the thinner cortical plate in newborn rats (Zechel et al., 2005; Table 1). Vasilev et al. (2016) demonstrated a short period of hypoxia at E14 or E18 impairs radial migration. Notably, hypoxia at E14, but not at E18, disturbed lateral dispersion of excitatory neurons in cortical minicolumns (Table 1). This finding indicates that the occurrence of neuronal migration defects due to prenatal hypoxia is timing-dependent. The fetal brain in earlier gestation period may be more susceptible to hypoxia-induced disturbance of neuronal migration. However, the relationship between the timing and severity of neuronal migration defect is still unclear, and thus more investigations are required.

One of the potential mechanisms underlying the impaired neuronal migration under prenatal hypoxia is the activation of lysophosphatidic acid (LPA) signaling. Herr et al. (2011) demonstrated that LPA signaling is activated by acute prenatal hypoxia in mice (Table 1). LPA is a lysophospholipid that activates LPA receptors and affects neurogenesis, neuronal migration, neuritogenesis, and myelination (Ishii et al., 2004). Notably, hypoxia-induced impaired neuronal migration was mitigated in E13 brain slices of Lpa1 receptor knockout mice exposed to hypoxia for 17 h. This observation suggests that defects of neuronal migration under hypoxia is mediated by Lpa1 receptor (Herr et al., 2011).

## Valproic Acid

Maternal use of valproic acid (VPA), an anti-epileptic drug, during pregnancy is closely associated with increased frequency of congenital malformation and neuropsychiatric disorders in human (Meador et al., 2013). A cohort study comparing the various anti-epileptic drugs that are prescribed to pregnant women found that the risks of fetal malformation increase in a dose-dependent manner. VPA exposure especially presents a greater risk of major congenital malformation compared to other antiepileptic drugs such as carbamazepine, lamotrigine, and phenobarbital (Tomson et al., 2011). VPA at daily doses of 1000 mg or higher is clearly associated with developmental abnormalities, thus frequent consumption of the VPA at reduced dose is recommended control epilepsy during pregnancy (Ornoy, 2009). Of note, the manifestation of autism is also strongly associated with prenatal exposure to VPA. Children who are exposed to VPA prenatally have a three-fold higher risk of developing autism (Christensen et al., 2013). Therefore, the mouse model of prenatal exposure to VPA has been widely used as one of non-genetic ASD models. In fact, these animals mimic many of ASD-like behaviors, such as social interaction deficits, repetitive self-grooming, digging, and deficiency in sensory processing (Roullet et al., 2010; Nicolini and Fahnestock, 2018).

The exact mechanism of how VPA treats epilepsy is not fully understood, yet proposed mechanisms include alteration of GABA levels, blockade of voltage-gated sodium channels (VGSCs) and inhibition of histone deacetylases (Rosenberg, 2007). All of which may contribute to abnormal brain development. For example, blocking or enhancing GABA_A_ receptor diminished or promoted the migration of MGE-derived interneurons into the cerebral cortex, respectively (Cuzon et al., 2006), demonstrating ambient level of GABA is important for stimulating the migrating neurons. Similarly, the activation of VGSCs induced Ca^2+^ signaling in GABA-mediated migration of oligodendrocyte precursor cells, and the knockdown of VGSCs by siRNA diminished intracellular Ca^2+^ level and reduced the migration (Tong et al., 2009). Thus, the blockage of VGSCs in neurons by VPA can lead to neuronal migration impairment via similar mechanism.

In addition, prenatal exposure to VPA during the peak of cortical neurogenesis in mice changes expressions of the genes that are associated with the cell migration, including BDNF and *Cxcr4*. This results in the mislocalization of excitatory neurons in the cerebral cortex in these animals (Almeida et al., 2014; Sakai et al., 2018; Table 1). It indicates that disturbance of BDNF/CXCL12/CXCR4 signaling is another potential mechanism underlying the deficiency of the neuronal migration by prenatal VPA exposure.

## Selective Serotonin Reuptake Inhibitor

Selective serotonin reuptake inhibitors (SSRIs) are commonly used to treat depression during pregnancy. The human imaging study demonstrated a significant increase of the gray matter volume in amygdala and insula along with increased connectivity in children exposed to SSRI prenatally (Lugo-Candelas et al., 2018). The expression of the genes involved in serotonin signaling starts during the early stage of fetal brain development, and the disruption of serotonin signaling pathway affects cell proliferation, differentiation, neuronal migration and network formation in various regions of the brain (Sodhi and Sanders-Bush, 2004).

The differential effects of SSRI depending upon the given dosage and/or timing is obscure. A study has shown that the children exposed to SSRIs in late pregnancy (>29 weeks) is strongly associated with increased risk of anxious or depressed behaviors (Lupattelli et al., 2018). Whereas another study has shown that the length of SSRI exposure rather than the timing crucially contributes to the outcome of the fetal development (Oberlander et al., 2008). Similarly, another study shows that extended SSRI exposure in prenatal period increases the risks of lower scores in psychomotor developmental index and behavioral rating scale in infancy (Casper et al., 2011).

Given that the polymorphisms of the genes encoding the serotonin transporters and receptors are strongly associated with neurodevelopmental disorders such as ASD (Anderson et al., 2002), both reduced and increased levels of serotonin signaling are proposed to cause devastating consequence in brain development. Consistent with this hypothesis, excessive activation of serotonin signaling disturbed the migration of pyramidal neurons in the cortex of the serotonin transporter (*Sert*) knockout (KO) mouse, in which serotonin accumulated extracellular and the activation of serotonin receptors was extended (Riccio et al., 2011; Table 1). The authors also demonstrated that excess activation of a subtype of serotonin receptor 5-HT_6_ that is expressed in the developing excitatory neurons disturbed the migration in the *Sert* KO cerebral cortex. In line with the hypothesis above, downregulation of 5-HT_6_ receptor also resulted in mislocalization of pyramidal neurons in the mouse cortex (Jacobshagen et al., 2014). Given that serotonin signaling also interacts with BDNF signaling, the migration may be disrupted by, at least in part, the similar mechanism observed in the cases of prenatal exposure to cocaine and VPA as described above (Sodhi and Sanders-Bush, 2004).

## Methylmercury

Exposure to a high level of heavy metal, such as methylmercury, is associated with neuropsychological problems. Methylmercury ingested by a pregnant woman is absorbed in the gastrointestinal tract and forms a placenta-crossing complex with L-cysteine (Broussard et al., 2002). Children exposed to methylmercury in the prenatal period show deficits in finger tapping speed, reaction time on a continuous performance task, and cued naming (Debes et al., 2006). A classic example of neurotoxicity due to prenatal methylmercury exposure is patients of Minamata disease. The brain development of these patients is severely compromised, resulting in ataxia, dysarthria, and tremor (Harada, 1978).

Similar to the cases of fetal exposure to alcohol described in previous section, mice that were exposed to methylmercury from postnatal day 6 to 9, the peak of neuronal migration in the cerebellum, showed a decrease in the frequency of spontaneous Ca^2+^ dynamics as well as impaired migration of cerebellar granular cells (CGCs) (Fahrion et al., 2012; Table 1). Notably, the migration defects were ameliorated by caffeine supplementation, which induces Ca^2+^ release by binding to ryanodine receptors in the brain and stimulating internal Ca^2+^ release and Ca^2+^ influx (Usachev et al., 1993; Fahrion et al., 2012). Additionally, a proteomic study of mouse embryo exposed to methylmercury reported a decrease in the phosphorylated form of cofilin in CGCs (Vendrell et al., 2010). LIM-kinase 1 (LMK-1)-mediated phosphorylation inactivates cofilin, by which prevents disassembly of actin filaments. Therefore, the change of phosphorylation ratio of cofilin would disrupt the actin dynamics in the filopodia of migrating CGCs (Yang et al., 1998; Fujimura et al., 2009; Fujimura and Usuki, 2012).

Chronic exposure to methylmercury, even at a very low dose of 0.1 mg/kg/day, affects neuronal migration in the developing rat cerebral cortex. Methylmercury suppresses protein expressions of Rac family small GTPase 1 (RAC1), Cell division cycle 42 (CDC42), and Ras homolog gene family, member A (RHOA), all of which are key players of radial migration in the developing brain (Govek et al., 2011; Guo et al., 2013; Table 1). Altogether, similar to alcohol, methylmercury changes the Ca^2+^ signaling and the expressions of genes or proteins that are closely linked to the control of cortical neuronal migration.

Another potential mechanism of methylmercury affecting the neuronal migration may be the serotonin signaling. Placental serotonin, which is synthesized from maternal tryptophan precursor, is required for neuronal migration during the forebrain development (Bonnin et al., 2011). Methylmercury not only inhibits cellular proliferation and migration, but also alters the expressions of oxidative stress-related genes such as superoxide dismutase 1 (SOD1) in human placenta-derived trophoblasts (Tucker and Nowak, 2018). The vulnerability of the trophoblasts to methylmercury suggests the potential effects on placental development (Hsiao and Patterson, 2012). Therefore, the maldevelopment of the placenta under exposure to methylmercury leads not only to the loss of protection for the fetus but also to disrupted production of the neurotransmitters required for proper neuronal migration.

## Glucocorticoids

Glucocorticoids (GCs) are a class of corticosteroids that are prescribed to pregnant women to promote lung maturation in fetuses when they are at risk of preterm delivery (Marciniak et al., 2011). Synthetic GC binds to the endogenous GC receptors in the fetal lung and promotes the maturation by increasing a production of the surfactant-associated proteins and phospholipids, both of which are crucial for stable lung function (Bolt et al., 2001).

The benefits of GC treatment for fetal lung maturation may come with a cost as studies show that the excess prenatal steroid exposure is associated with a wide spectrum of deficits in brain development, such as loss of hippocampal neurons, delayed myelination, and increased risk of neurodevelopmental disability (Velísek, 2005). Structural abnormalities including reduced cortical thickness in frontal cortex and rostral anterior cingulate cortex (rACC) were also evident in children who had been exposed to GC prenatally (Modi et al., 2001; Davis et al., 2013). Different dosages and timing of GC exposure may have differential effects on the developing brain. Treatment prior to mid-gestation may be well tolerated by the fetus without adverse effects due to the relatively high expression of a GC degrading enzyme, 11β-hydroxysteroid dehydrogenase (11β-HSD2), in the placenta and the fetal brain before mid-gestation (Diaz et al., 1998). In addition, the expressions of GC receptor transcripts start to arise in the pons, medulla, anterior hypothalamus, and spinal cord at mid-gestational stage of pregnancy (E12.5). Then gradually spread to the rest of brain regions such as neocortex in rats (Diaz et al., 1998) which may increase the fetal susceptibility to GC.

Animal studies demonstrate that neuronal migration defect by GC exposure is mediated by alterations in the expression of key neuronal migration genes. The administration of GC retarded the radial migration of excitatory neurons in fetal cerebral cortex. These neurons also showed increase in *Cald1* expression. Consistent with this, overexpression of *Cald1* led to impaired neuronal migration *in vitro* (Fukumoto et al., 2009; Table 1). *Cald1* encodes Caldesmon that negatively regulates the function of myosin II, an essential protein for the actin dynamics in the leading process of neuron. The upregulation of *Cald1* inhibits interactions between actin and myosin II in the radially migrating neurons, thereby disrupting the migration (Sobue and Fukumoto, 2010; Morita et al., 2012).

Interneurons in offspring of dams that underwent daily physical stress (restraint stress) exposure starting from E12 until birth show delayed migration without changes in the survival or proliferation (Table 1; Stevens et al., 2013). The expressions of transcription factors *dlx2* and *nkx2.1* in the fetal brains are significantly decreased as early as 24 h after the first stress exposure (Stevens et al., 2013). These transcription factors are important in determining cell fate and migration of interneuron (Nóbrega-Pereira et al., 2008). Overall, excess GC affects neuronal migration altering the expressions of the genes that directly and indirectly modulate the neuronal migration.

## Maternal Immune Activation

Maternal immune activation (MIA) is caused by an infection during pregnancy, and it is linked to increased risk of ASD and schizophrenia (Patterson, 2009). Maternal infection to human influenza virus during pregnancy resulted in ectopic cluster formation of Purkinje cells (PC) in the white matter of cerebellar lobules VI and VII in mouse, while the number of PC decreased in lobule VII (Shi et al., 2009; Table 1). A similar reduction in the number of PC in lobule VII was observed in mice born to mothers inoculated with an immunostimulant, poly(I:C) (Shi et al., 2009). These reports suggest that MIA disrupts the migration of the PC during cerebellar development. Given a subclass of small cytokines functions as migrational cues for newly generated neurons, the excess production of maternal cytokines is likely to trigger misguidance of neurons in the fetal brain under MIA (Du et al., 2014). MIA also altered the expressions of genes that are involved in the regulation of cell proliferation, migration, and axon guidance in the fetal brain (Oskvig et al., 2012; Lombardo et al., 2018). However, the contributions of each of such genes to the neuronal migration remain elusive.

## Molecular Mechanisms Shared by Different Types of Environmental Stress

**FIGURE 2:**
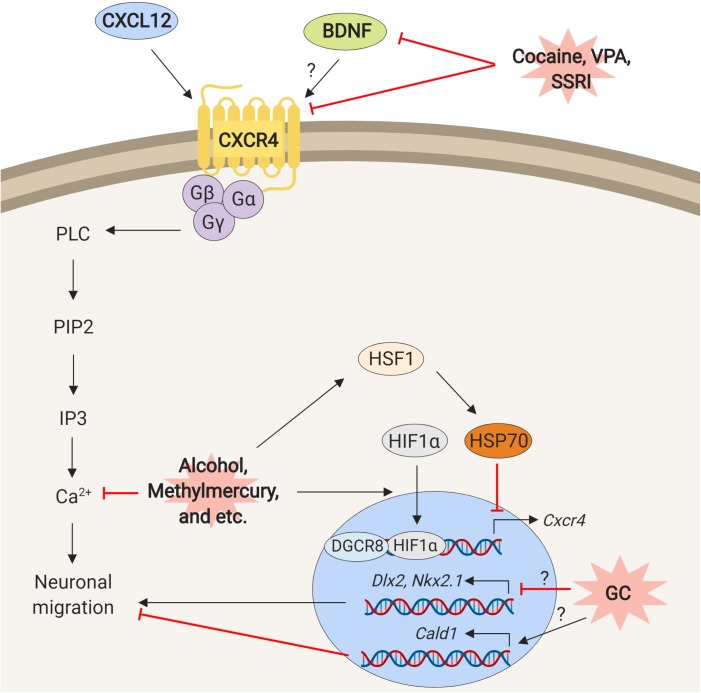
Schematic representation of mechanisms underlying neuronal migration defects elicited by exposure to environmental stress. The prenatal environmental stressors such as cocaine, VPA, SSRI, ethanol, methylmercury similarly, disturb interactions and functions of the molecules that are involved in the control of neuronal migration.

As described in previous sections, different types of prenatal environmental stress have common effects on the neuronal migration at the molecular level (Summarized in Figure 2). An example described above is the BDNF/CXCL12/CXCR4 signaling. Many of the chemicals and stress listed in this review alter the expressions of genes involved in this pathway. Interestingly, both excessive and insufficient expressions and activations of the genes in this signaling disturb the neuronal migration. Similarly, as described above, both up- and down-regulation of serotonin signaling disturb neuronal migration.

How does CXCR4/CXCL12 signaling control the neuronal migration? The clues may be found in the genetic disorder, 22q11.2 deletion syndrome (22q11.2DS). The mouse model of 22q11.2DS shows neuronal migration defects due to decrease of *Cxcr4* (Meechan et al., 2012). This phenotype is rescued by overexpression of the *Dgcr8* (Toritsuka et al., 2013). DGCR8 forms a complex with Drosha to process primary microRNA (pri-miRNA) and facilitates microRNA (miRNA) maturation (Gregory et al., 2004; Han et al., 2004). This finding suggests that the disturbance of the CXCL12/CXCR4 pathway may change miRNA-mediated regulation of the transcriptions that control neuronal migration (Sathyan et al., 2007).

The environmental stress may alter the expressions of genes in the CXCL12/CXCR4 pathway via a different route. Heat shock transcription factor 1 – hypoxia inducible factor-1 alpha (HSF1-HIF1α) pathway is a stress response signaling that is activated by various types of environmental stress in fetal brain (Hashimoto-Torii et al., 2014; Ishii et al., 2017; Torii et al., 2017), while HIF1α also directly transcribes *CXCR4* (Schioppa et al., 2003; Gabai et al., 2012). Thus the HIF1α may transcribe *CXCR4* in response to environmental stress (Ishikawa et al., 2009). On the other hand, overexpression of heat shock protein 70 (hsp70) that is transcriptionally regulated by HSF1 reduces *Cxcr4* expression (Sakurai et al., 2015).

Another common molecular trait shared by different types of environmental stress is the changes in expressions of genes that regulate the dynamics of the cytoskeleton. These changes happen partially through the activation of stress response signaling and the Ca^2+^ signaling (Kumada et al., 2006; Fahrion et al., 2012; Lombardo et al., 2018).

## Perspectives

Although animal models convey the mechanisms responding to environmental stress, what happens in the large human brain remains speculative. As rodents and humans do not share the identical mechanism of neuronal migration during the development of brain, studying brain development in a model that closely mimics human brain development is imperative (Semple et al., 2013). Therefore, the human pluripotent stem cell-derived three-dimensional organoid culture system, in which the cortical lamination and thick germinal zone are reproduced, may be a versatile tool to understand the molecular basis of neuronal migration defects in the human brain (Mason and Price, 2016; Wang, 2018). However, current protocols of the brain organoid system often require culture conditions that increase the levels of reactive oxidative stress (ROS). Thereby an optimal protocol that allows testing the effects of environmental stress in organoids without high ROS level is still needed. An especially intriguing question to be answered by using the organoid system will be whether the pathways commonly disrupted during neuronal migration by different types of environmental stressors are similarly, or differently regulated in human brain organoid in comparison to animal models.

## Author Contributions

HH and RK contributed to the writing and editing of the manuscript, and KH-T reviewed and edited the manuscript. All authors reviewed, discussed, and commented on the manuscript.

## Conflict of Interest Statement

The authors declare that the research was conducted in the absence of any commercial or financial relationships that could be construed as a potential conflict of interest.
